# Phased regulatory mechanism of sowing date-meteorological factor interaction on maize yield formation and resource use efficiency in Southwest China hilly region under climate warming conditions

**DOI:** 10.3389/fpls.2026.1889116

**Published:** 2026-07-22

**Authors:** Yonghong Wang, Fan Liu, Wanyuan Liu, Pijiang Yin, Xinglong Wang, Yuxin Zhou, Cailing Xu, Xiaolong Li, Dongju Feng, Fanlei Kong, Jichao Yuan

**Affiliations:** 1 College of Agronomy, Sichuan Agricultural University, Ministry of Agriculture, Crop Ecophysiology and Cultivation Key Laboratory of Sichuan Province, Chengdu, China; 2 Key Laboratory of Crop Ecophysiology and Farming System in Southwest China, Chengdu, China; 3 College of Life Science and Agri-Forestry, Southwest University of Science and Technology, Mianyang, China; 4 Agricultural and Rural Bureau of Zhongjiang County, Deyang, China

**Keywords:** climate change, grain yield, meteorological factor, resource use efficiency, sowing date, *Zea mays* L.

## Abstract

**Introduction:**

Climate warming has increased extreme weather events, threatening maize yield stability in Southwest China. Optimizing sowing date is crucial for matching meteorological resources with crop growth demands, yet the stage-specific regulatory mechanisms remain unclear.

**Methods:**

A five-year field experiment (2015–2016 and 2019–2021) with five sowing dates (March to May) was conducted at the Zhongjiang Experimental Station in the Sichuan Basin. Meteorological factors, grain yield, and its components were measured, and correlation analyses were performed.

**Results:**

The climate in the study area has warmed significantly over the past 35 years, with a substantial increase in the frequency of extreme heat events. Early sowing (SD1) significantly increased maize yield and enhanced its stability. For each day of sowing delay, dry matter accumulation decreased by 77.4 kg ha-1, leading to a yield reduction of 58.0 kg ha-1. The coefficient of variation for yield increased significantly for sowings in May, primarily attributed to the increased frequency of adverse weather conditions, such as high temperatures and heavy rain, during the pollination and grain-filling period. Different yield components were governed by dominant meteorological factors specific to key growth stages. Total dry matter accumulation was primarily driven by solar radiation (Sr), growing degree days (GDD) and diurnal temperature range (DTR) over the entire growth period. The kernel number per ear was sensitive to the maximum temperature (Tmax) during the 10 days before and after silking, with temperatures exceeding 33 ℃ causing significant reduction. The 1000-grain weight was positively regulated by Sr and GDD during the grain-filling period but negatively correlated with precipitation (for each 1 mm increase, the 1000-grain weight decreased by 0.063 g). With delayed sowing date, solar radiation and temperature utilization efficiency during the grain-filling period exhibited a linear decline.

**Discussion:**

This study identified late-March as the optimal sowing date for maize in the Sichuan Basin, as it achieved the best meteorological resources matching across all growth stages. The findings revealed the interaction mechanism of sowing date-meteorology-maize yield formation from a stage-specific perspective, providing a theoretical foundation and technical pathway for achieving high- and stable-yield in Southwest China under climate change.

## 1 Introduction

Maize (*Zea mays* L.) as the largest grain crop, plays a pivotal role in ensuring global food security (Kong et al., 2024). It serves not only a major feed crop but also an important industrial raw material, with extensive uses and high demand (Erenstein et al., 2022; Mushayi et al., 2025). In recent years, Chinese maize production has achieved remarkable results, with planting area reaching 44.74 million hectares and total output hitting 294.92 million tons by 2024 (National Bureau of Statistics of China, 2025: Accessed May 14, 2026). However, its production capacity cannot fully meet market demand, with imports from abroad reaching 13.64 million tons (General Administration of Customs of the People's Republic of China, 2025). Therefore, further increasing maize grain yields is of great significance for ensuring China food security, promoting livestock development and advancing rural revitalization.

Timely sowing is an effective measure to improve maize grain yield (Wang et al., 2025a). Adjusting the sowing date would affect the acquisition of meteorological resources such as solar radiation, temperature, and precipitation by maize during various growth stages, thereby influencing its growth, development and yield formation (Li et al., 2022). Timely sowing could align the spatiotemporal distribution of key meteorological resources with the physiological needs of the crop to achieve high yields (Tao et al., 2014). Grain yield was decreased due to delayed sowing dates, resulting in reductions in both ear kernel number and grain weight in temperate regions, with the decrease in grain weight being more pronounced than that in kernel number (Bonelli et al., 2016). Early-sown crops yield higher due to greater solar radiation (Sr) during the growing period (Wu et al., 2024). Sun et al. (2025) found that delaying sowing by 10 or 20 days beyond the optimal sowing date increased daily minimum temperature, shortened interval between sowing and jointing, thereby reducing grain nutritional quality and yield. Timely sowing also avoided natural disasters (e.g., high temperatures and droughts) (Lu et al., 2017; Xu et al., 2021), while ensuring the accumulated temperature required for maize growth, thereby guaranteeing high and stable yields (Rahimi-Moghaddam et al., 2018). Global warming in recent years is an indisputable fact (Alexandrov and Hoogenboom, 2000). In 2023, the global surface temperature was 0.55–0.60 °C higher than the 1991–2020 average and 1.35–1.54 °C higher than the 1850–1900 average (the period often used to represent pre-industrial conditions) (Blunden and Boyer, 2024). Over the past century, the warming rate in China has been 0.90–1.52 °C per 100 years, with a trend of 0.23 °C per decade over the last 50–60 years (Ding and Wang, 2016). Concurrent with climate warming, the frequency of extreme weather events (e.g., high temperatures and droughts) had significantly increased (Hu et al., 2023; Su et al., 2025), which had already substantially impacted the stability of global crop production (Kimball, 2016; Li et al., 2022). Under these new circumstances, optimizing sowing dates is more crucial for achieving high and stable maize yields.

China has a vast territory with diverse climatic and ecological conditions across regions, leading to varying optimal sowing dates for maize (Tao et al., 2014). A considerable amount of research has already been conducted on this topic (Chen et al., 2025; Huang et al., 2020; Li et al., 2022; Sun et al., 2025; Wu et al., 2024), particularly in the North China Plain. However, the suitable sowing window period for maize in these regions is relatively narrow, and large-scale sowing date studies are scarce. The relationship between maize grain yield formation and temperature-light resources needs further clarification under climate warming conditions. The stage-specific interactive mechanisms between sowing date, meteorological resource allocation, and yield component formation remain poorly understood. Southwest China, the third-largest maize-producing region in the country, is characterized by light and thermal resources, allowing for maize planting schedules from March to July (with March to May being the main period in Sichuan Basin). This provides greater scope for adjusting and optimizing sowing dates in this region. We hypothesize that the interaction between sowing date and meteorological factors improves the formation of important yield traits in maize by modulating the key meteorological factors at different growth stages. Accordingly, we conducted a five-year experiment with large-scale sowing dates in the Sichuan Basin. The objectives were: 1) To investigate the sowing-date effects on maize dry matter accumulation and yield components, and identify the local optimal sowing date; 2) to clarify the distribution of meteorological resources under different sowing dates and their matching patterns with maize growth requirements; and 3) to quantify the relationships between dry matter accumulation and yield components with key meteorological factors, identifying the critical meteorological factors during important growth stages. This study provides theoretical basis and practical guidance for optimizing sowing dates to achieve high- and stable-yields.

## 2 Materials and methods

### 2.1 Experiment site and design

**Figure 1:**
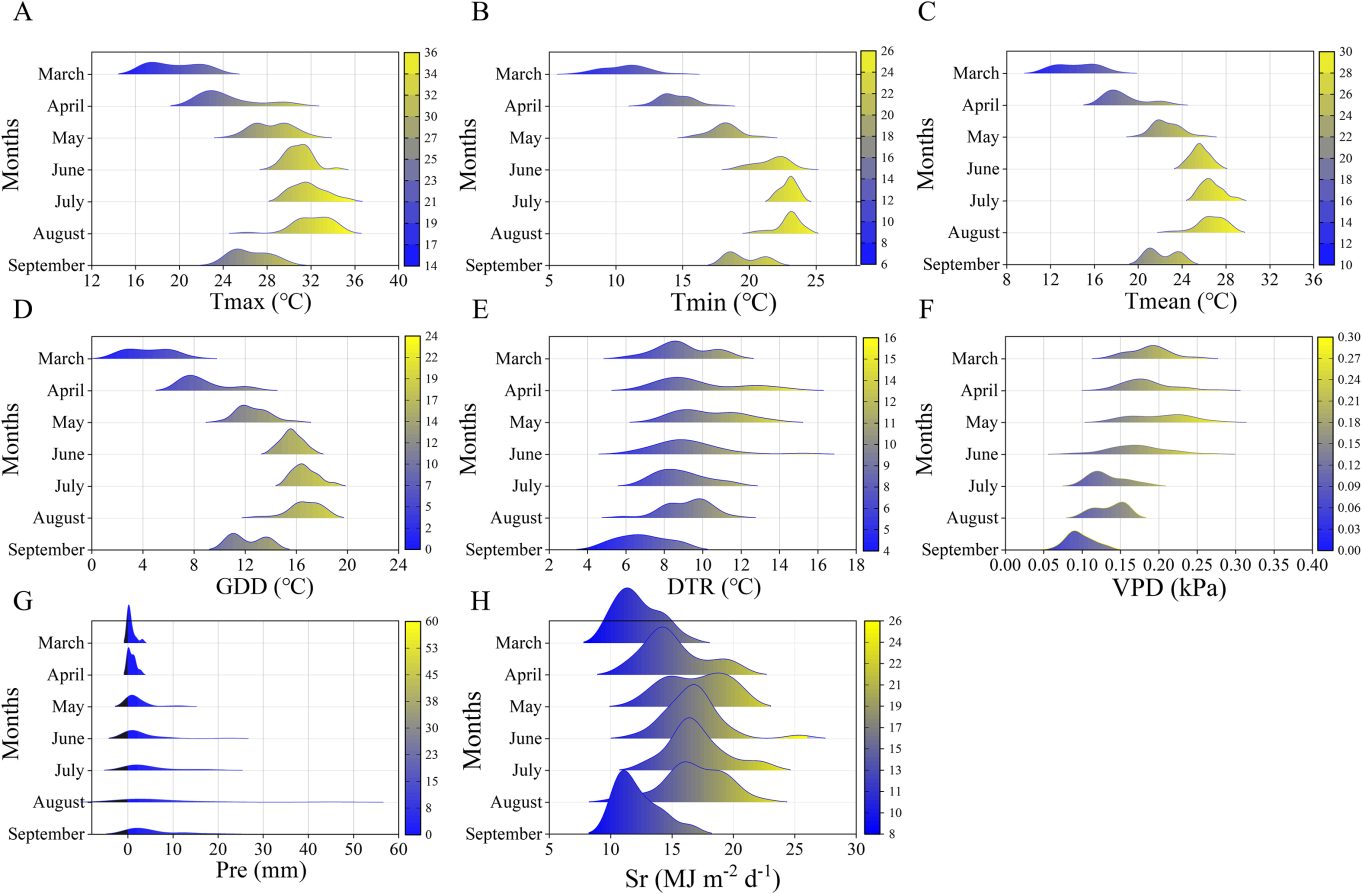
Meteorological data during maize growing season in 2015–2016 and 2019-2021. **(A)** Tmax, daily maximum temperature; **(B)** Tmin, daily minimum temperature; **(C)** Tmean, daily mean temperature; **(D)** GDD, growing degree days; **(E)** DTR, daily diurnal temperature range; **(F)** VPD, monthly vapor pressure deficit; **(G)** Pre, monthly precipitation; **(H)** Sr, solar radiation.

The field experiment was conducted from 2015–2016 and 2019–2021 at Zhongjiang Experimental Station of Sichuan Agricultural University (31°03′ N, 104°68′ E). Detailed information regarding the soil properties of the experimental site could be found in reference Wang et al. (2025b). The region experienced a subtropical monsoon climate. The daily maximum temperature (Tmax), minimum temperature (Tmin), mean temperature (Tmean), growing degree days (GDD), daily diurnal temperature range (DTR), daily vapor pressure deficit (VPD), solar radiation (Sr) and precipitation (Pre) during the maize growing seasons across the five years were presented in **Figure 1**.

The hybrid tested was ZhengHong 505 (ZH505), widely cultivated locally, with seeds supplied by Sichuan Zhenghong Biotechnology Co., Ltd. The experiment employed a single-factor randomized block design with five sowing dates (the first on March 25 or 26, with subsequent sowings at 15-day intervals, the actual sowing dates, together with the corresponding emergence, silking, and physiological maturity dates across the five experimental years, are presented in **Supplementary Table 1** and Wang et al. (2025b)), with three replicates for each treatment. The planting density was approximately 49,500 plants ha^-1^. In 2015-2016, each plot area was 17.6 m^2^ (5.5 m × 3.2 m) with a row spacing of 80 cm. To further enhance the representativeness and reliability of the experiment, the plot size was increased to 27 m^2^ (6 m × 4.5 m) with a 75 cm row spacing in 2019-2021. Applying 112.5 kg N ha^-1^, 72 kg P_2_O_5_ ha^-1^ and 90 kg K_2_O ha^-1^ before sowing. An additional top-dressing of 112.5 kg N ha^-1^ was applied at V12. Field management practices followed local high-yield protocols and were kept uniform across all plots.

### 2.2 Meteorological data

Meteorological data were provided by the Zhongjiang County Meteorological Bureau. Growing degree days (GDD) represented the accumulated difference between the daily mean temperature and a specified base temperature (Tb, i.e., the biological minimum temperature) (**Equation 1**) (Anandhi, 2016; McMaster and Wilhelm, 1997). Diurnal temperature range (DTR) was the difference between the daily maximum and minimum temperatures (**Equation 2**). Killing degree days (KDD) quantified the cumulative heat stress when the daily maximum temperature exceeded the upper optimal threshold for maize growth (32 °C) (**Equation 3**) (Liu et al., 2021; Qiao et al., 2025; Wu et al., 2024). Vapor pressure deficit (VPD) was the key parameter indicating the difference between the actual and saturation vapor pressure which was calculated as described by Lou et al. (2025) (**Equations 4**–**6**). The formulas for calculating these parameters are as follows:

(1) $$\text{GDD}=\sum \left(\frac{\text{T}\max+\text{T}\min}{2}-\text{Tb}\right)$$

(2) DTR=Tmax−Tmin

(3) $$\text{KDD}=\{\begin{array}{ll} \sum_{\text{i}=1}^{\text{n}}\text{T}\max-Thigh & ,\;if\;Tmax>Thigh\\ \sum_{\text{i}=1}^{\text{n}}0 & ,\;if\;Tmax\leq Thigh \end{array}$$

(4) $$\text{VPD}=e_{0}-e_{a}$$

(5) $$e_{0}=0.6108\exp(\frac{17.27\times \text{Tmean}}{\text{Tmean}+237.3})$$

(6) $$e_{a}=e_{0}\left(\frac{\text{RHmean}}{100}\right)$$

where Tb denotes the base temperature for maize growth (≥ 10 °C), Tmax is the daily maximum temperature (°C), and Thigh is the upper temperature limit for optimal maize growth (32 °C). e_0_ is the daily mean saturation vapor pressure (kPa), e_a_ is the daily mean actual vapor pressure (kPa), and RHmean is the daily mean relative humidity.

### 2.3 Growth stages

Growth stages were accurately recorded for each treatment. Emergence (VE) was defined as the date when the first true leaf was unfolded in over 60% of seedlings. Silking (R1) was characterized as the date when more than 50% of plants had silks protruding 3 cm beyond bract. Physiological maturity (R6) was determined as the date when the milk line of the grains in the ear middle part of 50% of plants had disappeared, with the black layer appearing at its base.

### 2.4 Dry matter accumulation

At R1 and R6, five healthy and uniformly growing and representative plants were selected from each plot. Following the methods described by Dong et al. (2024), they were first oven-dried at 105 °C for 45 minutes to deactivate enzymes and then dried at 80 °C to constant weight before weighing.

### 2.5 Solar radiation and temperature utilization efficiency

Daily solar radiation (Sr, MJ m^-2^ d^-1^) (**Equation 7**) and active accumulated temperature (Tb, d °C) were calculated following methods described by He et al. (2020) and Wen et al. (2018), respectively. Subsequently, the solar radiation utilization efficiency (RUE, MJ m^-2^) (**Equation 8**) and temperature utilization efficiency (TUE, kg ha^-1^ °C^-1^) (**Equation 9**) were determined (Guo et al., 2025).

(7) $$\text{Sr}={\text{R}}_{\text{a}}\left(\text{a}+\text{b}\frac{\text{n}}{\text{N}}\right)$$

(8) $$\text{RUE}=\frac{\text{Y}}{\sum Sr}$$

(9) $$\text{TUE}=\frac{\text{Y}}{\sum \text{Tb}}$$

where Sr is the solar radiation, Ra is the extraterrestrial radiation, n is the actual sunshine duration (hour), N is the maximum possible sunshine duration (He et al., 2020), and a and b are empirical coefficients set at 0.25 and 0.50, respectively, for western regions (Yuan et al., 2018). Y represents the dry matter accumulation per unit area (kg ha^-1^). ∑Sr is the total solar radiation (MJ m^-2^) during a specific growth period, and ∑Tb is the sum of daily mean temperatures ≥ 10 °C (°C d) during that period, i.e., the effective accumulated temperature.

### 2.6 Grain yield and its components

At physiological maturity, all ears from each plot were harvested to determine grain yield with 14% moisture content. Twenty representative ears per plot were selected using the average ear weight method to investigate the ear kernel number, 1000-grain weight (Wu et al., 2023). The annual coefficient of variation (CV) was used to assess yield stability (Han et al., 2020) (**Equation 10**).

(10) $$\text{CV}=\frac{\text{standard deviation}}{\text{Mean}}\times 100\%$$

### 2.7 Statistical analysis

Data processing was performed using Microsoft Excel. Analysis of variance was performed using IBM SPSS Statistics 25 (SPSS Inc, Illinois, USA). The least significant difference test (LSD) was applied to examine the significance of differences between treatments at the 5% level. Correlation and regression analyses were conducted using Origin (OriginLab Corporation, USA). Contribution rates between indicators were calculated based on the residual sum of squares from regression equations. Variation partitioning analysis (VPA) was performed using the “vegan” package (version 2.6) in R 4.1.2 (R Core Team, New Zealand) to further investigate climatic effects on yield components. The random forest (RF) algorithm was implemented using the “randomForest” package (version 4.7) was used to evaluate the contribution of meteorological factors to yield components. Figures were generated using R 4.1.2 and Origin. Linear regression analysis was performed to quantify the relationships between sowing date (day of year) and meteorological variables during different growth stages.

## 3 Results

### 3.1 Climate changes during the maize growing season from 1990 to 2025 in study site

**Figure 2:**
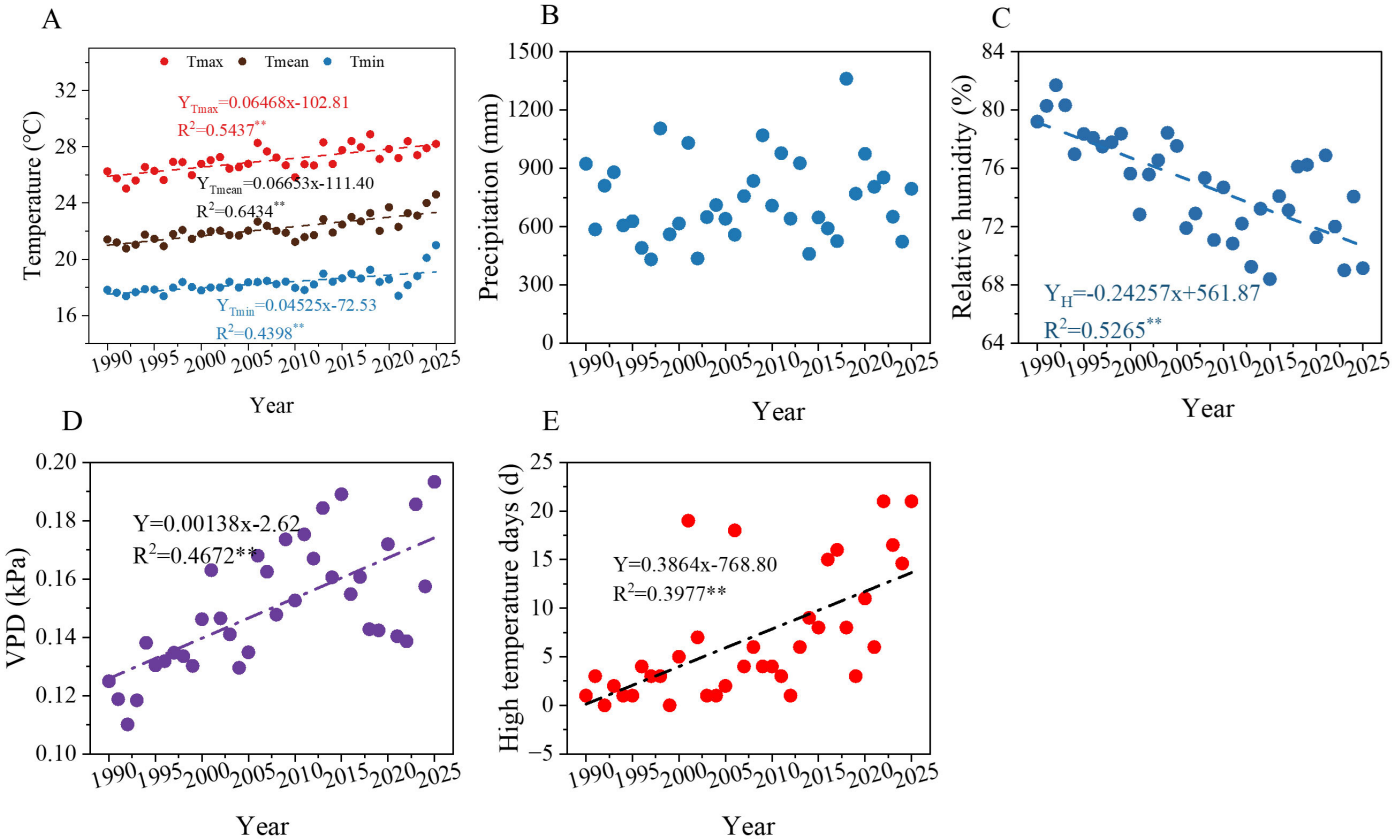
The trends of main meteorological factors during the maize growth season in Zhongjiang County from 1990 to 2025. **(A)** Daily maximum, mean, and minimum temperatures; **(B)** annual precipitation; **(C)** relative humidity; **(D)** vapor pressure deficit (VPD); **(E)** high temperature days (daily maximum temperature > 35 ℃ during June to July). Temperature and relative humidity were averaged during March to September; precipitation was the total value during March to September. Tmax, Tmin, and Tmean represented the daily maximum, minimum, and mean temperature, respectively.

From 1990 to 2025, The daily maximum temperature (Tmax), minimum temperature (Tmin), and mean temperature (Tmean) during the maize growing season (from March to September) in Zhongjiang County, the study site, showed a significant upward trend, with annual increases of 0.06, 0.07, and 0.05 °C, respectively (**Figure 2A**). There were no significant changes in precipitation (Pre) distribution (**Figure 2B**). Relative humidity (RH) showed a significant decreasing trend, with a decline of 0.24% per year (**Figure 2C**), while vapor pressure deficit (VPD) increased by 0.001 kPa per year (**Figure 2D**). The number of days with Tmax exceeding 35 °C during June–July increased at a rate of 0.39 days per year (**Figure 2E**). These indicated that the climate in the study area has warmed significantly over the past 35 years, with a substantial increase in the frequency of extreme heat events.

### 3.2 Meteorological conditions during maize growing season and its response to sowing dates

During the experimental period (2015-2016, 2019-2021), the temperatures (Tmax, Tmin and Tmean) from March to September (locally maize growing season) exhibited a pattern of initial increase followed by a decrease. The Tmax, Tmin, Tmean and GDD, which peaked in July and August, were relatively low from March to May, and began to decline in September (**Figures 1A–D**). DTR continuously increased from March to May and then decreased rapidly from June to September (**Figure 1C**). The Sr followed a trend similar to temperature, but with relatively smaller variations from June to August (**Figure 1G**). VPD was also higher from April to June, leading to increased crop evapotranspiration demand (**Figure 1E**). Pre gradually increased after March, reaching the peak daily average of 7.70 mm in August (**Figure 1F**).

Altering the sowing date changed main meteorological conditions (**Table 1**). During entire maize growth period (VE-R6), GDD and KDD initially increased and then decreased with sowing date delay, while temperature (Tmax, Tmin and Tmean), RH, and Pre gradually increased. Conversely, Sr, DTR, and VPD gradually decreased. Delaying sowing date by one day increased Tmax, Tmin, Tmean, RH and Pre by 0.0311 °C, 0.0372 °C, 0.0354 °C, 0.0933%, and 2.88 mm, respectively, while decreasing Sr, DTR and VPD by 5.61 MJ m^-2^, 0.0069 °C and 0.0006 kPa, respectively (**Supplementary Figure 6**). During the kernel number mainly formation period (Pre-_silking10_-Post-_silking10_, i.e., 10 days before to after silking), with delaying sowing date, Tmin and Tmean gradually rose, DTR initially decreased and then increased, RH first increased and then slightly decreased, VPD showed the opposite trend, while Sr and Tmax fluctuated slightly from SD1 to SD3 and then rose. During the grain weight formation period (Post-_silking10_-R6), Sr, temperature, DTR and Pre first increased and then decreased with sowing date delay, whereas RH and VPD remained relatively stable.

**Table 1:** Effects of sowing date on meteorological factors at different growth stages of maize (5-year average).

Growth stage	Sowing date	Sr (MJ m-2)	Tmax (°C)	Tmin (°C)	Tmean (°C)	DTR (°C)	RH (%)	VPD (kPa)	Pre (mm)	GDD (°C d)	KDD (°C d)
VE-R6	SD1	1966.6 ± 92.71	29.69 ± 0.96	20.31 ± 0.42	24.99 ± 0.67	9.36 ± 0.67	69.94 ± 6.08	0.18 ± 0.04	342.05 ± 68.23	1630 ± 41.64	69.16 ± 31.42
	SD2	1919.54 ± 83.72	30.43 ± 0.89	20.99 ± 0.37	25.72 ± 0.63	9.42 ± 0.58	71.97 ± 5.41	0.17 ± 0.03	398.55 ± 130.8	1645.08 ± 45.27	91.46 ± 29.89
	SD3	1867.98 ± 66.53	30.88 ± 0.83	21.65 ± 0.45	26.27 ± 0.61	9.23 ± 0.4	73.33 ± 3.82	0.16 ± 0.02	462.27 ± 157.84	1691.32 ± 21.74	152.33 ± 76.39
	SD4	1766.54 ± 95.15	31.12 ± 0.36	22.01 ± 0.21	26.75 ± 0.53	9.02 ± 0.41	74.62 ± 3.69	0.15 ± 0.02	508.85 ± 202.82	1644.44 ± 50.57	119.03 ± 36.5
	SD5	1626.46 ± 117.36	31.66 ± 0.73	22.57 ± 0.38	27.11 ± 0.51	9.05 ± 0.46	75.56 ± 3.56	0.14 ± 0.02	501.59 ± 213.08	1577.92 ± 102.42	116.59 ± 36.99
Pre-silking 10-Post-silking 10	SD1	331.7 ± 26.31	30.58 ± 1.75	21.4 ± 1.05	26.01 ± 1.33	9.2 ± 1.1	68.81 ± 7.49	0.19 ± 0.04	61.17 ± 31.3	328.56 ± 34.41	12.8 ± 7.56
	SD2	333.35 ± 23.67	30.8 ± 1.8	21.94 ± 0.84	26.38 ± 1.31	8.88 ± 1.06	72.49 ± 6.69	0.16 ± 0.04	71.43 ± 29.42	313.92 ± 25.84	15.22 ± 14.13
	SD3	315.37 ± 31.19	30.66 ± 1.51	22.48 ± 0.64	26.56 ± 1.02	8.18 ± 1.09	77.04 ± 1.8	0.14 ± 0.01	60.66 ± 30.55	336.24 ± 33.03	12.66 ± 9.96
	SD4	332.09 ± 25.1	31.68 ± 1.24	22.82 ± 1.01	27.15 ± 1.06	8.9 ± 0.66	77.66 ± 3.85	0.13 ± 0.02	76.36 ± 42.15	329.06 ± 20.43	19.44 ± 11.72
	SD5	293 ± 148.03	32.45 ± 1.56	22.95 ± 0.67	27.79 ± 0.94	9.32 ± 1.37	76.3 ± 7.19	0.14 ± 0.05	103.56 ± 62.03	339.56 ± 18.32	30.78 ± 16.52
Post-silking 10-R6	SD1	638.59 ± 54.16	31.5 ± 0.91	22.88 ± 0.58	27.2 ± 0.73	8.62 ± 0.48	77.82 ± 5.52	0.14 ± 0.03	172.78 ± 70.73	651.04 ± 42.42	43.14 ± 19.34
	SD2	672.76 ± 66.66	32.09 ± 1.02	23.16 ± 0.61	27.64 ± 0.79	8.95 ± 0.71	77.37 ± 5.04	0.13 ± 0.03	231 ± 129.26	661.6 ± 22.26	53.88 ± 19.99
	SD3	695.22 ± 69.33	32.5 ± 1.18	23.23 ± 0.49	27.86 ± 0.73	9.22 ± 0.99	76.67 ± 5.25	0.14 ± 0.03	277.88 ± 170.87	668.98 ± 26.76	112.23 ± 69.08
	SD4	660.23 ± 29.68	31.63 ± 1.6	22.62 ± 0.83	27.95 ± 1.18	9.21 ± 0.88	76.38 ± 5.19	0.14 ± 0.03	303.36 ± 194.23	634.34 ± 16.79	70.29 ± 19.55
	SD5	554.37 ± 69.02	31.62 ± 1.31	22.71 ± 0.6	27.18 ± 0.99	8.89 ± 0.63	77.78 ± 4.25	0.13 ± 0.02	271.4 ± 166.45	563.8 ± 65.54	53.03 ± 27.6

VE-R6, from emergence to physiological maturity; Pre-_silking10_-Post-_silking10_, 10 days before to 10 days after silking; Post-_silking10_-R6, from 10 days after silking to physiological maturity. SD1-SD5, the first to fifth sowing date. Sr, solar radiation; Tmax, Tmin, Tmean and DTR, daily maximum temperature, minimum temperature, mean temperature and diurnal temperature range; RH, relative humidity; VPD, vapor pressure deficit; Pre, precipitation; GDD and KDD, growing degree days and killing degree days. Values are presented as means ± standard deviation (n = 5 years).

**Figure 3:**
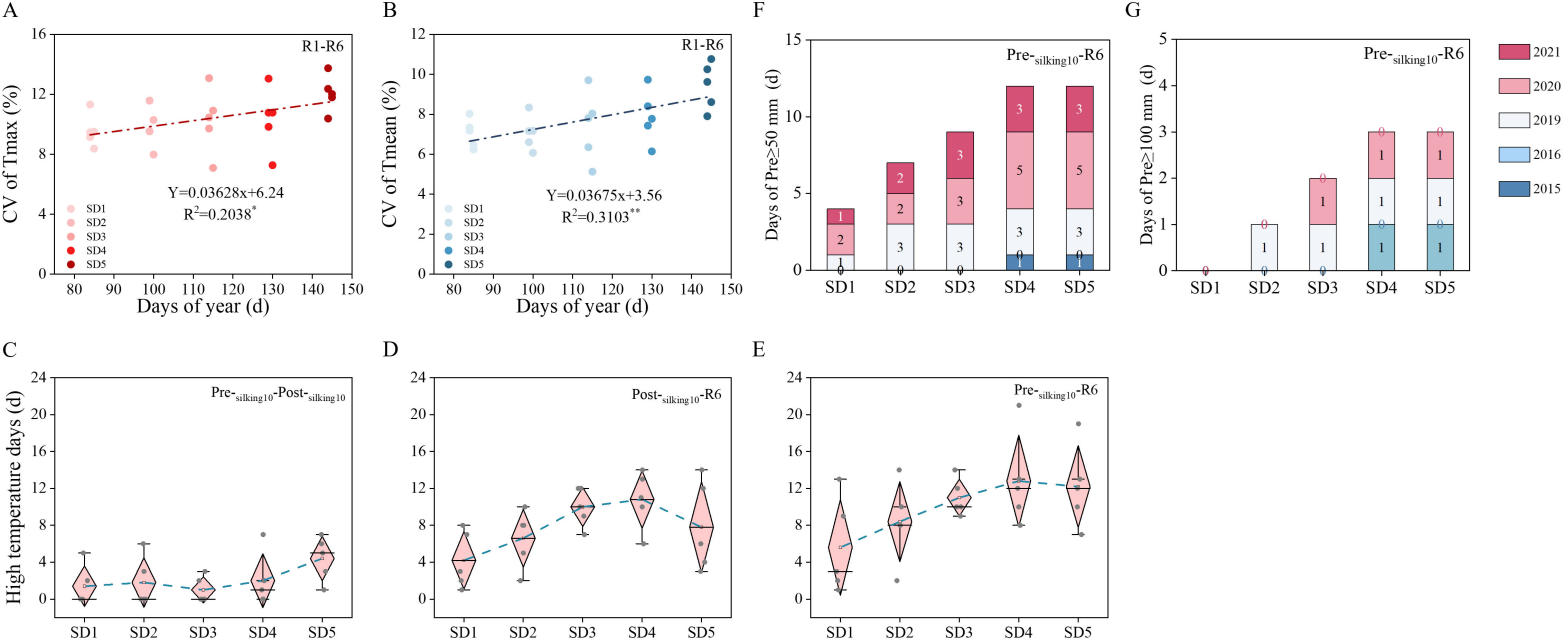
Temperature variability **(A, B)**, frequency of high temperatures (Tmax ≥ 35 °C) **(C-E)** and heavy rainfall events (daily precipitation ≥ 50 mm or 100 mm) **(F, G)** during the late growth period. CV, Coefficient of variation. Days of year, sowing date, the number of days after January 1^st^. Pre-_silking 10_- Post-_silking 10_, Pre-_silking 10_-R6, Post-_silking 10_-R6, and R1-R6, representing the growth period from 10 days before silking to 10 days after silking, from10 days before silking to maturity, and from10 days after silking to maturity, and from silking to maturity, respectively. SD1-SD5, the first to fifth sowing date. The difference significance at 0.05, 0.01, and 0.001 levels were marked by *, **, and ***.

Adjusting the sowing date also significantly affected the annual stability of meteorological factors and frequency of extreme weather events during maize’s major growth period (**Figure 3**). The CV for temperature (Tmax and Tmean) during R1-R6 gradually increased with later sowing date, with SD5 exhibiting higher temperature fluctuations (**Figures 3A, B**). The high-temperature days and frequency of heavy rain events (especially the period from 10 days before silking to R6 which are the main formation period of ear kernel number and 1000-grain weight) showed an increasing trend with delayed sowing (**Figures 3C–G**). Late sowing (SD4 and SD5) resulted in greater temperature variability, more high-temperature days and more frequent occurrence of heavy rain, which may adversely affect maize growth and grain yield.

### 3.3 Effects of sowing date on maize growth period, dry matter accumulation, grain yield and its components

**Figure 4:**
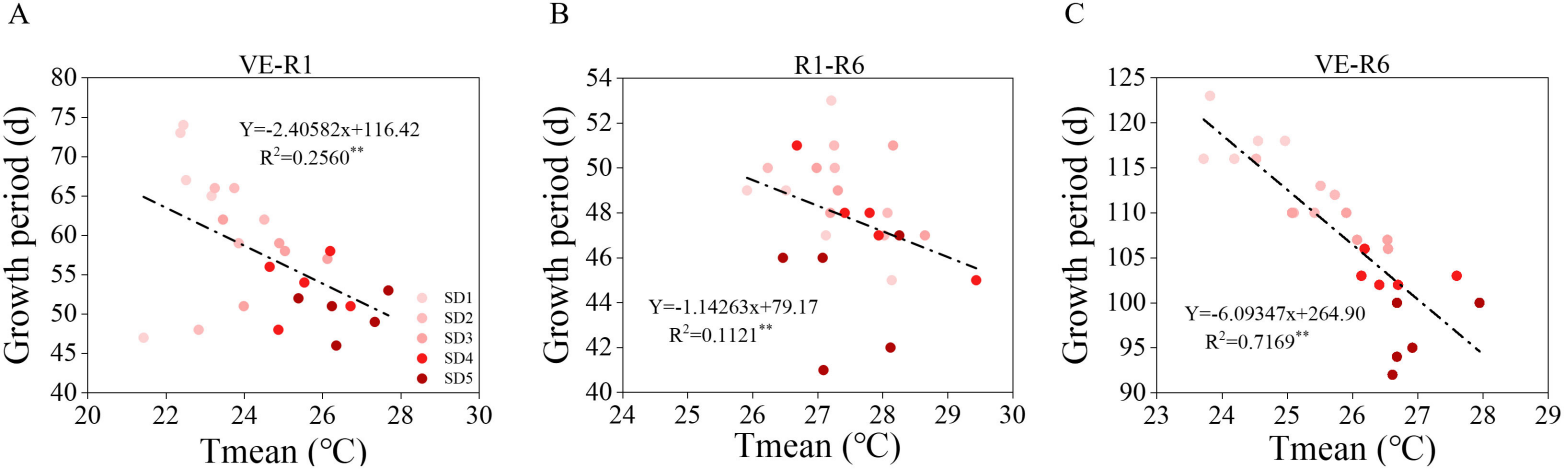
The relationship of growth period with daily mean temperature. **(A)** VE-R1, from emergence to the silking; **(B)** R1-R6, from silking to physiological maturity; **(C)** VE-R6, from emergence to physiological maturity. The trends significant at 0.05, 0.01, and 0.001 levels were marked by *, **, and **.

With delaying sowing date, maize growth period was gradually shortened due to rising temperatures, particularly in VE-R1, while R1-R6 initially showed a slight increase before a rapid reduction (Wang et al., 2025b). For each 1 °C increase in daily mean temperature (Tmean), the durations of VE-R1, R1-R6, and VE-R6 shortened by 2.41, 1.14 and 6.09 days, respectively (**Figures 4A-C**). Delaying sowing date by 1 day reduced the VE-R1 and R1-R6 periods by 0.295 and 0.066 days, respectively (**Supplementary Figure 1**).

**Figure 5:**
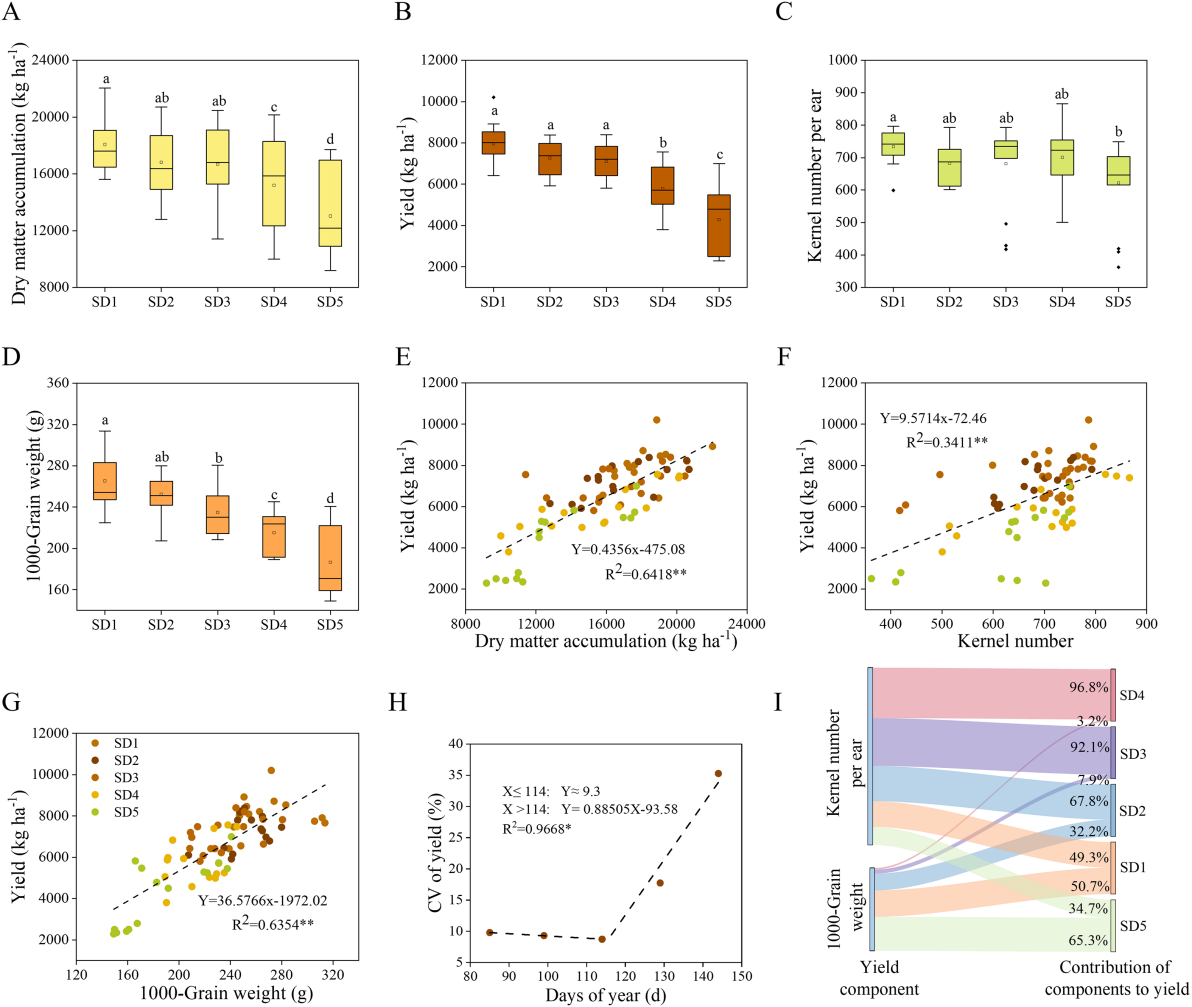
Effects of sowing date on dry matter accumulation, yield composition **(A-D)**, the relationship of yield with its components **(E-G)**, the coefficients of variation in yield **(H)** and its sources of variation **(I)** for different sowing date. SD1-SD5, the first to fifth sowing date. Different letters indicate a significant (*P<0.05*) difference between different sowing date. Days of year, sowing date, the number of days after January 1^st^. The difference significance at 0.05, 0.01 and 0.001 levels were marked by *, ** and ***.

Adjusting sowing date significantly influenced maize grain yield and its components, as well as dry matter accumulation (DMA) (**Figures 5A–D**). With delaying sowing date, DMA, ear kernel number, 1000-grain weight and grain yield gradually decreased, particularly when sowed in late May. Yield was highest for SD1 (early sowing, 7955.87 kg ha^-1^) and lowest for SD5 (late sowing, 4277.03 kg ha^-1^), with SD5 yielding 46.2% and 26.0% less than SD1 and SD4, respectively.

Sowing date primarily affected maize grain yield and its components by influencing DMA. Yield was significantly positively correlated with DMA, 1000-grain weight and kernel number. For each 1 kg ha^-1^ increase in DMA, grain yield increased by 0.436 kg ha^-1^ (**Figure 5E**). For each 1 g increase in 1000-grain weight, grain yield increased by 36.6 kg ha^-1^ (**Figure 5G**). For each additional kernel number, grain yield increased by 9.57 kg ha^-1^ (**Figure 5F**). Linear regression analysis further revealed that for each day of sowing date delay, grain yield decreased by 58.0 kg ha^-1^, DMA decreased by 77.4 kg ha^-1^, ear kernel number decreased by 1.39 kernels per ear, and 1000-grain weight decreased by 1.30 g (**Supplementary Figures 2A–D**). The total growth period (VE-R6) was significantly positively correlated with DMA and yield, with each day of growth period reduction corresponding to a 221.7 and 166.3 kg ha^-1^ decrease respectively (**Supplementary Figures 2E, F**).

Differences existed not only in grain yield but also in its CV across sowing dates. Sowing in April or earlier (SD1-SD3) resulted in lower coefficient of variation in yield across years, whereas sowing in May led to a rapid increase in CV (increased by 0.89% for each day of delay) (**Figure 5H**).

Analysis of the primary sources of grain yield variation under different sowing dates revealed that for SD1, the contributions of ear kernel number and 1000-grain weight were each close to 50% (**Figure 5I**). With delaying sowing date, the contribution of kernel number gradually increased, reaching 96.8% for SD4 (contributing 1.11 percentage points more for each day of delay). However, this contribution dropped sharply to 34.7% for SD5, while the contribution of 1000-grain weight showed the opposite trend. Therefore, increasing ear kernel number for sowings before early May (SD4 and earlier) and increasing 1000-grain weight for sowings after mid-May (SD5) were the primary pathways to improving maize yield.

### 3.4 Relationships between dry matter accumulation and yield components with meteorological factors

**Figure 6:**
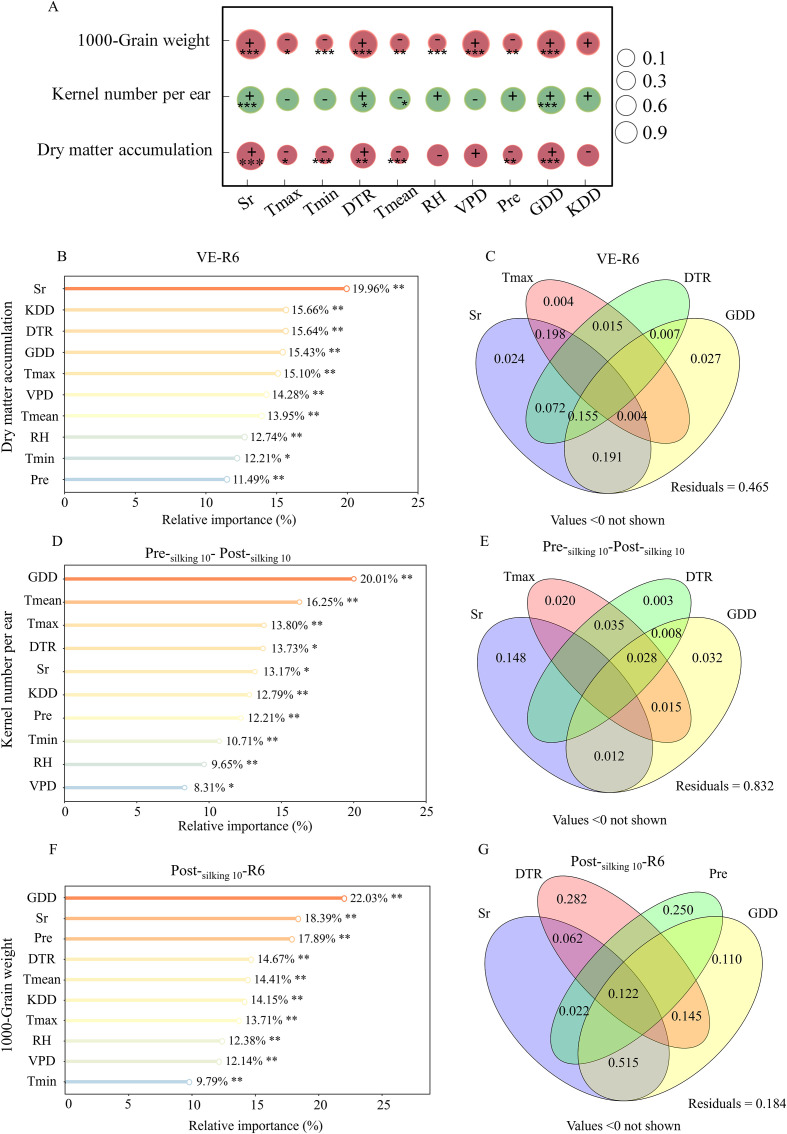
Meteorological factors influencing dry matter accumulation and yield components, and their contributions. Correlation coefficients with meteorological factors of the entire growth period **(A)**; Relative importance of meteorological factors derived from random forest analysis **(B, D, F)**; Contributions of primary meteorological factors obtained through Variation partitioning analysis **(C, E, G)**. Sr, solar radiation; Tmax, Tmin, Tmean and DTR, daily maximum temperature, minimum temperature, mean temperature and diurnal temperature range; RH, relative humidity; VPD, vapor pressure deficit; Pre, precipitation; GDD and KDD, growing degree days and killing degree days. The difference significance at 0.05, 0.01, and 0.001 levels were marked by *, **, and ***.

**Figure 7:**
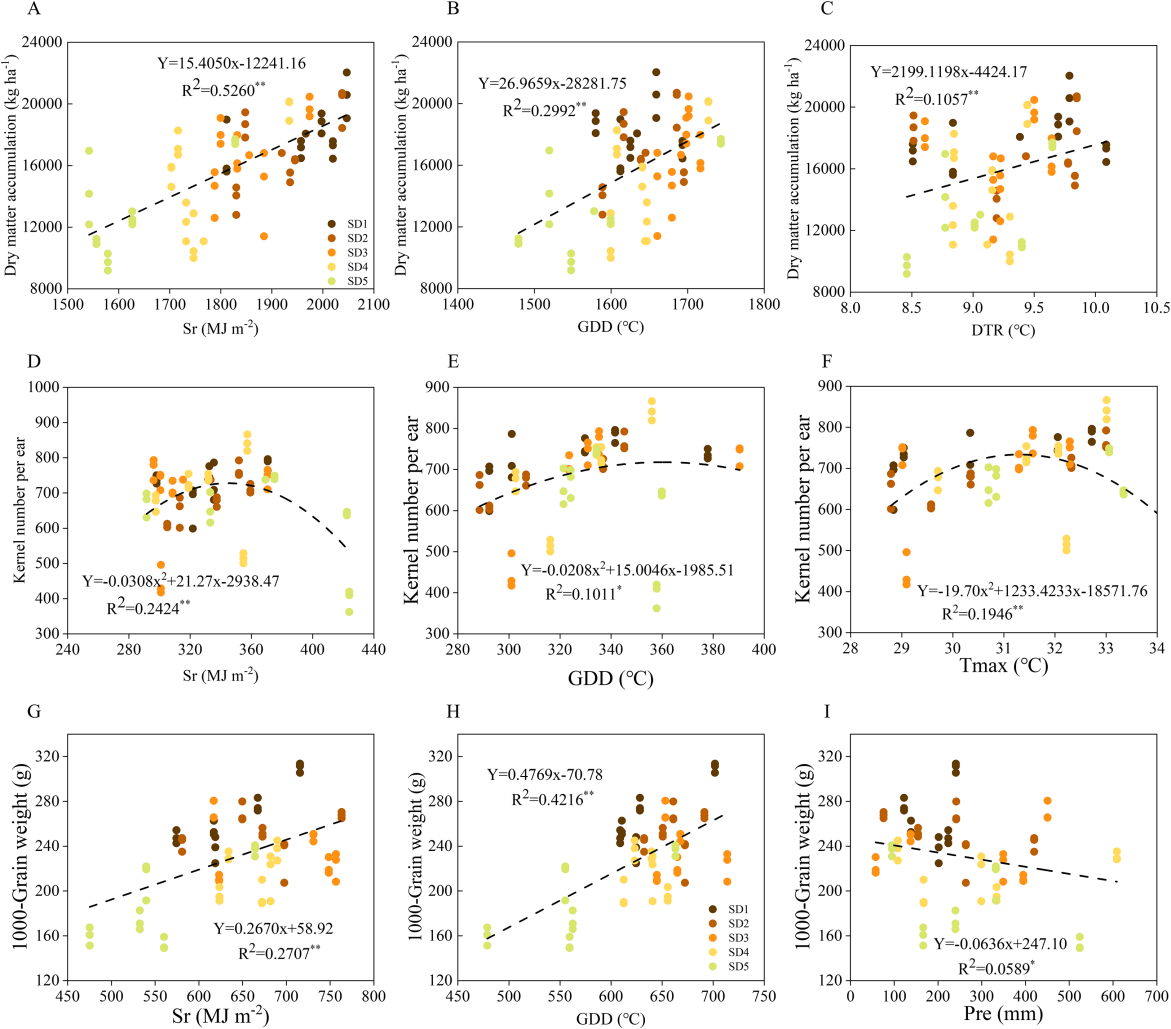
Relationship between dry matter accumulation and yield components with key meteorological factors. The meteorological factors correlated with dry matter accumulation are those during VE-R6 **(A-C)**, and that affecting ear kernel number are those during Pre-_silking 10_ to Post-_silking 10_ **(D-F)**, and while that affecting 1000-grain weight are those from post-_silking 10_ to R6 **(G-I)**. Sr, solar radiation; GDD, growing degree days; DTR, diurnal temperature range; Tmax, daily maximum temperature; Pre, precipitation. The difference significance at 0.05, 0.01, and 0.001 levels were marked by *, **, and ***.

Meteorological factors significantly impacted maize DMA and yield components (**Figure 6**). DMA was significantly positively correlated with Sr, DTR and GDD during VE-R6, and significantly negatively correlated with Tmin, Tmean and Pre (**Figure 6A**). Regression analysis showed that for each 1 MJ m^-2^ increase in Sr during VE-R6, DMA and yield increased by 15.4 kg ha^-1^ and 6.22 kg ha^-1^ respectively (**Figure 7A**, **Supplementary Figure 3A**). For each 1 °C increase in Tmean, DMA decreased by 807.95 kg ha^-1^ (**Supplementary Figure 3B**). For each 1 °C d increase in GDD, DMA and yield increased by 26.97 and 15.42 kg ha^-1^, respectively (**Figure 7B**, **Supplementary Figure 4**). Random forest analysis (RF) indicated that Sr, KDD, DTR and GDD during VE-R1 had greater influence on DMA (**Figure 6B**). Variation partitioning analysis (VPA) showed that these four factors collectively explained 53.5% variation, with the interaction between Sr and GDD, as well as Sr and Tmax, contributing the most, followed by that between GDD and DTR (**Figure 6C**). Regression analysis showed that DMA was significantly positively correlated with Sr, GDD and DTR (**Figures 7A–C**).

Ear kernel number was significantly positively correlated with Sr and GDD, and significantly negatively correlated with Tmean during VE-R6 (**Figure 6A**). RF analysis identified GDD, Tmean, Tmax, DTR and Sr during Pre-_silking10_-Post-_silking10_ as the primary factors influencing ear kernel number (**Figure 6D**). VPA indicated that GDD, Tmax, DTR and Sr explained 16.8% of the variation in ear kernel number, with Sr the largest contributor (**Figure 6E**). Regression analysis revealed that ear kernel number had convex quadratic relationships with Sr, GDD and Tmax during Pre-_silking10_-Post-_silking10_, suggesting optimal values of these factors maximizing ear kernel number (**Figures 7D–F**). Notably, a Tmax exceeding 33 °C would lead to a rapid decline in ear grain number.

The 1000-grain weight was significantly positively correlated with Sr, DTR, GDD and VPD, and significantly negatively correlated with Tmax, Tmin, Tmean, RH and Pre during VE-R6 (**Figure 6A**). RF analysis identified GDD, Sr, Pre and DTR during the grain-filling period (Post-_silking 10_-R6) as the key meteorological factors influencing 1000-grain weight (**Figure 6F**). VPA showed that these four factors collectively explained 81.6% variation, with the interaction between GDD and Sr contributing the most at 51.5%, while DTR and Pre also had substantial individual contributions (**Figure 6G**). Regression analysis showed that 1000-grain weight was significantly positively correlated with Sr and GDD, and significantly negatively correlated with Pre (**Figures 7G–I**). 1000-grain weight was also positively correlated with VPD during the VE-R6, with 675.95 g increase for every 1 kPa increase in VPD (**Supplementary Figure 5A**). This may be attributed to the synchronous increase in Sr and VPD (for every 1 kPa increase in VPD, Sr increased by 2576.8 MJ m^-2^) (**Supplementary Figure 5B**).

### 3.5 Effects of sowing date on RUE and TUE

**Figure 8:**
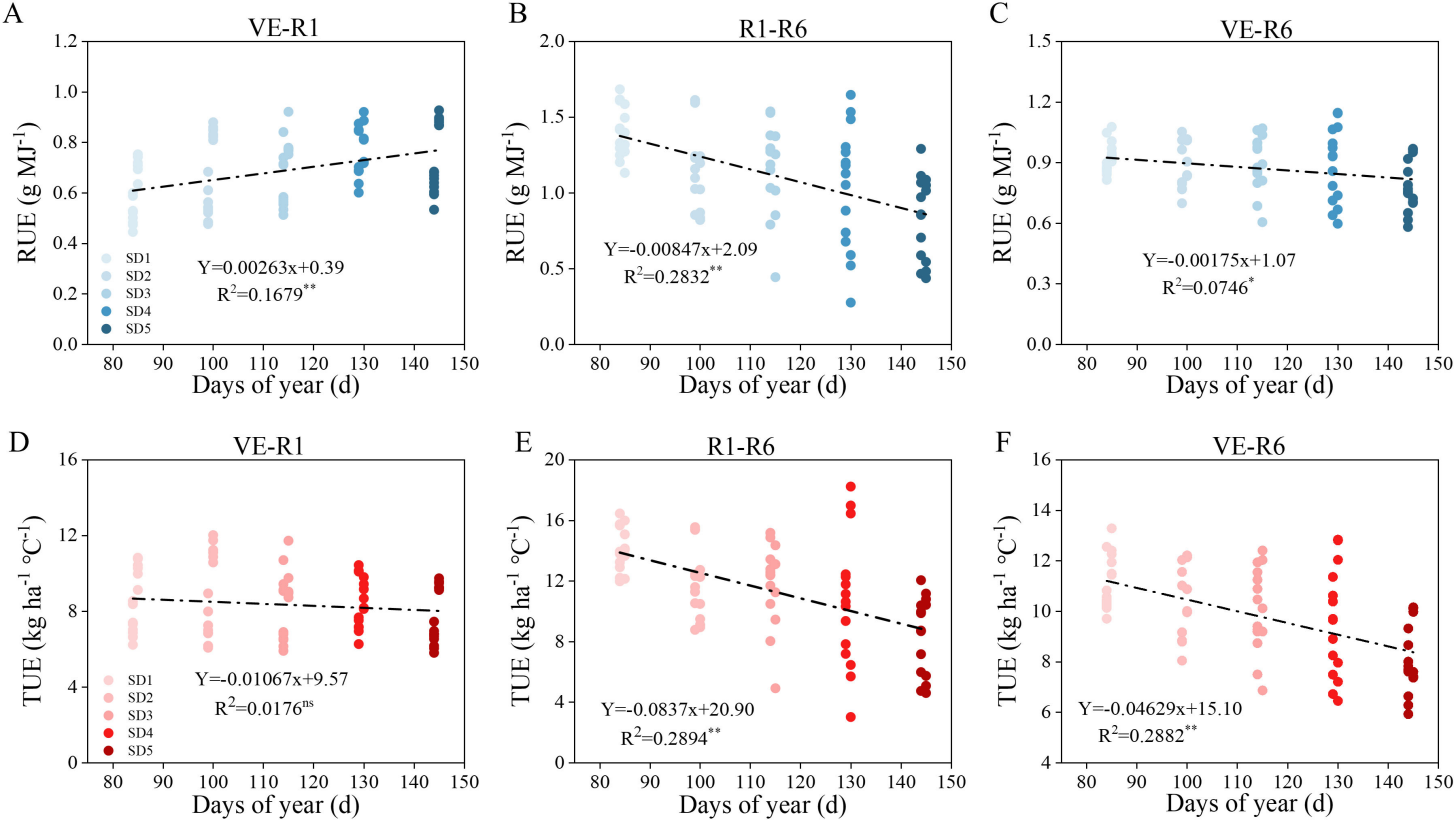
Relationship between sowing date and RUE and TUE during different growth periods. Days of year, sowing date, the number of days after January 1^st^. RUE, radiation use efficiency; TUE, temperature use efficiency. VE-R1, from emergence to the silking **(A, D)**; R1-R6, from silking to maturity **(B, E)**; VE-R6, from emergence to maturity **(C, F)**. The difference significance at 0.05 and 0.01 levels were marked by * and **, ns indicates not significant.

Sowing date, by affecting DMA, also significantly influenced RUE and TUE (**Figure 8**). Delayed sowing tended to increase RUE during VE-R1 but significantly decreased both RUE and TUE during R1-R6 and VE-R6. For each day of sowing date delay, RUE and TUE during R1-R6 decreased by 0.0085 g MJ^-1^ and 0.084 kg ha^-1^ °C^-1^, respectively, while those for VE-R6 decreased by 0.0018 g MJ^-1^ and 0.0463 kg ha^-1^ °C^-1^, respectively. Timely early sowing can enhance DMA, thereby improving RUE and TUE.

## 4 Discussion

### 4.1 Timely early sowing date achieves high- and stable-yield

Numerous experimental studies and production practices have demonstrated that sowing at the optimal date is fundamental for achieving high-yield of crops, as it can improve photosynthetic efficiency and utilization efficiency of solar radiation and temperature (He et al., 2020). Maize, a thermophilic crop (Ma et al., 2022), is typically sown in spring and summer in China, with a general recommendation for timely early sowing (Long et al., 2025; Yuan et al., 2012). Timely early sowing extended the growth period and photosynthetic duration, and increased the accumulation of photosynthetic products, thereby boosting maize yield (Sun et al., 2025; Zhu et al., 2022). Delayed sowing date led to higher temperatures during the early growth stages, accelerated development, shortened the dry matter accumulation period, limited the time for leaf area establishment, and reduced the maximum leaf area index (Rahmani et al., 2016). In Northeast China, delaying sowing date for each day shortened the total growth period by 0.1% and reduced potential yield by 0.6% (Zhu et al., 2022), corresponding to a yield loss of approximately 150 kg ha^-1^ (Xin and Tao, 2019). Under warm conditions, shifting from the normal to late sowing date might cause yield losses exceeding 20% (Dadrasi et al., 2024). However, earlier sowing was not always better. The relationship between maize yield and sowing date often followed a convex quadratic function, with both excessively early and late sowing potentially leading to yield reduction (Zhang et al., 2025b). Excessively early sowing might reduce yield due to insufficient rainfall during the vegetative stage (Li et al., 2024). Late sowing increased the risk of exposing maize to heat-temperature stress during critical reproductive stages (e.g., silking and grain-filling) or low-temperature stress during late grain-filling, leading to reductions in ear kernel number and 1000-grain weight (Dadrasi et al., 2024; Guo et al., 2022). However, some studies in the North China Plain suggested that delaying sowing date effectively reduced the probability of encountering high temperatures at silking, and improved solar radiation utilization during grain-filling, thereby increasing grain weight and yield (Li et al., 2022).

This five-year field experiment in Sichuan Basin hilly region demonstrated that maize yield gradually decreased with sowing date delayed after late March (especially after late April), with average yield reduction of 58.0 kg ha^-1^ per day of delay (**Supplementary Figure 2A**). This was associated with reduced dry matter accumulation (DMA), delaying sowing date for each day DMA decreased by 77.41 kg ha^-1^ (**Supplementary Figure 2B**). And the reduction in DMA was likely related to the shortened growth period, as they exhibited a highly significant positive correlation. For each day the growth period shortened, DMA decreased by 221.70 kg ha^-1^ (**Supplementary Figure 2E**). The shortening of the growing season is further related to rising temperatures (**Figure 4D**). Therefore, the primary reason for higher-yield under timely early sowing date was the relatively cooler temperatures, slower growth, longer growth duration and consequently greater accumulation of photosynthetic products. Regarding yield components, delayed sowing date significantly reduced both 1000-grain weight and ear kernel number, with a greater impact on 1000-grain weight. Each day of delayed sowing resulted in a decrease of 1.30 g in 1000-grain weight and 1.39 in ear kernel number (**Supplementary Figures 2C, D**).

The seasonal distribution and inter-annual fluctuation of temperature and precipitation were key factors affecting maize yield, with adverse weather (e.g., high temperature and heavy precipitation) explaining over 50% of yield variation (Su et al., 2025). Notably, timely early sowing not only achieved high yield but also ensured yield stability. The coefficient of variation (CV) for yield increased linearly for sowings after mid-April (**Figure 6H**). This might be attributed to the following factors: 1) Delayed sowing date led to higher and more unstable temperatures during the grain-filling period (**Table 1**), with CV for both Tmax and Tmean increased linearly, accompanied by a rise in the frequency of extreme high-temperature days (over 35 °C) (**Figures 3A–E**). Temperatures exceeding 35 °C were generally known to reduce maize pollen viability, affecting pollination and grain set (Rattalino Edreira et al., 2014; Liu et al., 2024), and could also impair photosynthesis, reducing the photosynthetic assimilates accumulation (Wang et al., 2023). Delayed sowing date increased the occurrence of strong winds and heavy rainfall events during the late growth stages (**Figures 3F, G**), which were considered significant contributors to lodging (Kong et al., 2024; Wei et al., 2025; Zhang et al., 2021, 2025c), consequently leading to increased field lodging rate (Wang et al., 2025b).

### 4.2 Key meteorological factors influencing yield

Meteorological conditions were among the important factors influencing maize yield variations under different sowing dates (Simon et al., 2023; Rajii et al., 2025; Wu et al., 2024). Sr and temperature were generally considered the primary meteorological factors affecting maize yield (Cao et al., 2024; Li et al., 2022; Niu et al., 2024; Zhou et al., 2017). Sr was the energy source for photosynthesis, and increased Sr leads to more photosynthetic products, with the two exhibiting a significant positive correlation. In this study, for each 1 MJ m^-2^ increase in Sr of total growth season, DMA increased by 15.4 kg ha^-1^ (**Figure 7A**), and yield increased correspondingly by 6.22 kg ha^-1^ (**Supplementary Figure 3A**). In the North China Plain, a 1 MJ m^-2^ increase in radiation enhanced kernel number by 8%-12%. Delayed sowing date might lead to insufficient solar radiation during the grain-filling period (Li et al., 2022), reducing RUE. The contributions of Sr to yield were 63.1% and 86.4% during the periods from 15 days before to 15 days after silking and from silking to harvest, respectively (Wu et al., 2024).

Temperature is a key environmental factor regulating plant growth and development, influencing physiological metabolism through its effects on enzyme activity, etc (Moore et al., 2021; Zhang et al., 2025a, 2023; Zhao et al., 2021). Suitable temperatures enhanced photosynthetic efficiency and assimilated accumulation, thereby improving crop productivity (Rattalino Edreira et al., 2014; Moore et al., 2021). However, high temperatures increased respiratory consumption and accelerated developmental rates, which were detrimental to DMA (Jian et al., 2024). In this study, Tmean, Tmax and Tmin were all significantly negatively correlated with total DMA, kernel number and 1000-grain weight. For each 1 °C increase in Tmean, total DMA decreased by 807.95 kg ha^-1^ (**Supplementary Figure 3B**). Simon et al. (2023) also observed this negative correlation between yield and temperature in the Transylvanian Plain. Temperatures that were too high or too low might reduce pollen viability, affecting fertilization and grain set, thus lowering kernel number (Li et al., 2025; Xu et al., 2024). In this experiment, the relationship between ear kernel number and Tmax during the 10 days before and after silking followed a quadratic function, with values below 30 °C and especially exceeding 33 °C causing a more pronounced reduction (**Figure 7F**). Temperatures between 33 and 36 °C during the pre-blooming and post-blooming periods of maize, reduce the CO_2_ exchange rate by ≈17%, the growth rate by 17–29%), the grain number by 7–45%) (Neiff et al., 2016). Growing degree days (GDD) over growth period also significantly influenced maize growth and development, and GDD during the active dry matter accumulation period and the late dry matter accumulation period could explain the majority yield variation (Chen et al., 2025). Studies suggested that delaying sowing date might shorten the total GDD, leading to insufficient accumulated heat and affecting yield, particularly for late-maturing hybrids (Li et al., 2022; Zhu et al., 2022). However, findings on the relationship between yield and GDD at different stages were inconsistent. Some studies reported the significant positive correlation between yield and pre-anthesis GDD, but a highly significant negative correlation with post-anthesis GDD (Zhang et al., 2024). Others founded a significant negative correlation between GDD and accumulated biomass during the VE-V12, but a significant positive correlation during the V12-R6 (Cao et al., 2024). Our study showed that GDD during VE-R6 and Post-_silking 10_-R6 were positively correlated with total DMA and 1000-grain weight, respectively. For each 1 °C increase in GDD, total DMA and 1000-grain weight increased by 27.0 kg ha^-1^ and 0.477 g, respectively (**Figures 7B, H**). Therefore, increasing GDD during the total seasonal and grain-filling period enhanced maize yield. Specifically, yield increased significantly by 15.42 and 19.64 kg ha^-1^ for each 1 °C increase in GDD during VE-R6 and Post-_silking 10_-R6, respectively (**Supplementary Figure 4**).

Furthermore, DTR was another thermal condition affecting plant growth (Sharma et al., 2023). Typically, higher daytime temperatures favor photosynthesis, while lower nighttime temperatures helped reduce respiratory consumption; thus, a larger DTR was conducive to dry matter accumulation (Niu et al., 2024). Summer maize yield was positively correlated with DTR across the entire growth period (Liu et al., 2025). This study found that for each 1 °C increase in the mean DTR during VE-R6, DMA increased by 2199.1 kg ha^-1^ (**Figure 7C**). However, Zhou et al. (2017) reported that during the silking-maturity period, the maximum grain growth rate occurred at 7.1 °C DTR. When the Tmin< 20.7 °C and DTR exceeded 7.1 °C, the grain growth rate decreased, leading to reduced grain weight.

In addition to temperature and light, other meteorological factors such as Pre also co-regulate plant growth and development (Xie et al., 2022). Some studies suggested that excessive Pre reduced yield, with kernel number showing a significant negative correlation with Pre during the silking-grain formation period (Liang et al., 2021). Conversely, insufficient Pre (drought) can also severely impact crop yield (Li et al., 2024). Delayed sowing date might misalign the crop growth period with the rainy season, leading to either insufficient or excessive total Pre during grain-filling and lowering water utilization efficiency (Feng et al., 2022). Excessive Pre during the tasseling-milking stage led to a decrease in 1000-grain weight and a significant yield reduction (Liu et al., 2025). In this study, Pre during post-_silking 10_-R6 was negatively correlated with 1000-grain weight. For each 1 mm increase in Pre, the 1000-grain weight decreased by 0.064 g (**Figure 7I**). VPD was another major factor influencing maize yield in China, with every 0.1 kPa increase in VPD reducing maize yield by 15% (Yu et al., 2022). However, in our experiment, VPD during VE-R6 was positively correlated with both dry matter accumulation and 1000-grain weight (**Figure 6A**). This may be related to the local conditions of high RH and low VPD (less than 0.2 kPa, **Table 1**), as too low VPD inhibits stomatal opening and suppresses transpiration (Cernusak et al., 2019). Furthermore, it was found that Sr is synchronous upward with VPD (**Supplementary Figure 5A**). The Sichuan Basin is classified as a region with low Sr (Liu et al., 2023). Increasing Sr can boost DMA and thereby enhance yield.

Regarding the primary meteorological factors affecting maize growth and yield formation, most studies pointed to Sr and temperature (Sun et al., 2025; Wu et al., 2024; Zhou et al., 2017), while others emphasize Pre and GDD (Lu et al., 2017; Wang et al., 2025b). This study indicated that the key meteorological factors influencing different growth stages and yield-forming components varied. Total DMA was primarily regulated by Sr, GDD, and DTR during VE-R6. Sr, GDD and Tmax during the pollination and grain set period (Pre-_silking 10_-Post-_silking 10_) were the main factors affecting ear kernel number, while Sr, GDD and Pre during the grain-filling period (Post-_silking 10_-R6) were the main factors influencing 1000-grain weight (**Figure 6**). This study also quantified the relationships between yield components and the key meteorological factors during its principal formation stages (**Figure 7**), which could provide support for optimizing sowing dates in maize production to achieve favorable meteorological conditions across all growth stages, thereby improving yield.

## 5 Conclusion

This study systematically analyzed the seasonal variation patterns of meteorological factors in maize and established the quantitative relationships between yield-forming components and key meteorological factors. It was found that timely early sowing extended the growth period, increased dry matter accumulation, and enhanced both maize yield and its stability. The regulatory effects of meteorological factors on yield components exhibited distinct stage-specific characteristics. In the hilly region of the Sichuan Basin, adjusting maize sowing date to late-March could effectively avoid unfavorable climatic events (e.g., high temperatures and heavy rainfall) during the late-growth stages, achieve optimal coupling between the supply of meteorological resources during the critical growth stages and crop physiological demand. These finding deepened the understanding of the meteorological response mechanisms underlying maize yield formation. It provided a solid theoretical basis and practical guidance for formulating adaptive cultivation strategies for maize high yielding in Southwest China under climate change.

## Data Availability

The raw data supporting the conclusions of this article will be made available by the authors, without undue reservation.
