# Supplementary material for: Phased regulatory mechanism of sowing date-meteorological factor interaction on maize yield formation and resource use efficiency in Southwest China hilly region under climate warming conditions
Source: Front Plant Sci. 2026 Jul 22;17:1889116. doi: 10.3389/fpls.2026.1889116 (PMC13437340; doi:10.3389/fpls.2026.1889116)
Supplement: Supplementary file 1 [file SupplementaryFile1.docx]

Supplementary Material

1 **Supplementary Figures and Table**

For more information on Supplementary Material and for details on the different file types accepted, please see here.

1.1 **Supplementary Figures**

**Supplementary Figure 1.** Relationships between sowing dates and days of the growing season. VE-R1, from emergence to the silking; R1-R6, from emergence to physiological maturity. The trends were significant at 0.05, 0.01 and 0.001 levels were marked by *, ** and ***.

**Supplementary Figure 2.** Relationships between sowing dates and yield **(A)**, dry matter accumulation **(B)**, kernel number per ear **(C)**, 1000-grain weight **(D)**. Relationships of dry matter accumulation **(E)**, yield **(F)** and growth period (VE-R6). The trends were significant at 0.05, 0.01 and 0.001 levels were marked by *, ** and ***.

**Supplementary Figure 3.** Relationships between Solar radiation (Sr) and yield **(A)**. Relationships between mean temperature (Tmean) and dry matter accumulation **(B)**. Sr and Tmean during the VE-R6 growth stage. The trends were significant at 0.05 and 0.01 levels were marked by * and **.

**Supplementary Figure 4.** Relationships between growing degree days (GDD) and yield during the stages of the grain-filling period **(A)** and the entire growth period **(B)**. Post_-silking 10_-R6, from post-silking 10 to physiological maturity; VE-R6, from emergence to physiological maturity. The trends were significant at 0.05 and 0.01 levels were marked by * and **.

**Supplementary Figure 5.** Relationships between Solar radiation (Sr) and vapor pressure deficit (VPD) **(A)** and relationships between vapor pressure deficit (VPD) and 1000-Grain weight **(B)**. VE-R6, from emergence to physiological maturity. The trends were significant at 0.05 and 0.01 levels were marked by * and **.

**Supplementary Figure 6.** Relationships between days of year (DOY) and key meteorological variables (Tmax, Tmin, Tmean, RH, Pre, Sr, DTR, VPD and GDD) across VE-R6 growth stage **(A-I)**. VE-R6, from emergence to physiological maturity. The trends were significant at 0.05 and 0.01 levels were marked by * and **, ns indicates not significant.

**1.2 Supplementary Table**

**Supplementary Table1: Sowing periods and growth stages (five years)**

| Year |  | Sowing Date | Emergence | Sliking | Physiological maturity |
| --- | --- | --- | --- | --- | --- |
|  | SD1 | 26-Mar | 31-Mar | 6-Jun | 25-Jul |
|  | SD2 | 10-Apr | 14-Apr | 15-Jun | 5-Aug |
| 2015 | SD3 | 25-Apr | 2-May | 29-Jun | 17-Aug |
|  | SD4 | 10-May | 16-May | 9-Jul | 26-Aug |
|  | SD5 | 25-May | 1-Jun | 22-Jul | 1-Sep |
|  | SD1 | 26-Mar | 31-Mar | 6-Jun | 25-Jul |
|  | SD2 | 10-Apr | 14-Apr | 15-Jun | 5-Aug |
| 2016 | SD3 | 25-Apr | 2-May | 29-Jun | 17-Aug |
|  | SD4 | 10-May | 16-May | 9-Jul | 26-Aug |
|  | SD5 | 25-May | 1-Jun | 22-Jul | 1-Sep |
|  | SD1 | 26-Mar | 2-Apr | 15-Jun | 3-Aug |
|  | SD2 | 9-Apr | 16-Apr | 21-Jun | 10-Aug |
| 2019 | SD3 | 24-Apr | 30-Apr | 1-Jul | 18-Aug |
|  | SD4 | 9-May | 15-May | 10-Jul | 26-Aug |
|  | SD5 | 24-May | 30-May | 21-Jul | 1-Sep |
|  | SD1 | 25-Mar | 7-Apr | 11-Jun | 3-Aug |
|  | SD2 | 9-Apr | 17-Apr | 18-Jun | 7-Aug |
| 2020 | SD3 | 24-Apr | 30-Apr | 26-Jun | 15-Aug |
|  | SD4 | 9-May | 15-May | 5-Jul | 25-Aug |
|  | SD5 | 24-May | 30-May | 18-Jul | 2-Sep |
|  | SD1 | 25-Mar | 3-Apr | 11-Jun | 28-Jul |
|  | SD2 | 9-Apr | 17-Apr | 18-Jun | 5-Aug |
| 2021 | SD3 | 24-Apr | 29-Apr | 27-Jun | 17-Aug |
|  | SD4 | 9-May | 13-May | 10-Jul | 26-Aug |
|  | SD5 | 24-May | 29-May | 22-Jul | 6-Sep |

Note: SD1-SD5, the first to fifth sowing date.
